# PRMT1 Confers Resistance to Olaparib via Modulating MYC Signaling in Triple-Negative Breast Cancer

**DOI:** 10.3390/jpm11101009

**Published:** 2021-10-08

**Authors:** Wen-Jing Hsu, Cheng-Hsun Chen, Yu-Chu Chang, Chia-Hsiung Cheng, Ying-Huei TsaI, Cheng-Wei Lin

**Affiliations:** 1 Department of Biochemistry and Molecular Cell Biology, School of Medicine, College of Medicine, Taipei Medical University, Taipei 110, Taiwan; d119110001@tmu.edu.tw (W.-J.H.); b614105024@tmu.edu.tw (C.-H.C.); yuchuc@tmu.edu.tw (Y.-C.C.); chcheng@tmu.edu.tw (C.-H.C.); b101106067@tmu.edu.tw (Y.-H.T.); 2 Graduate Institute of Medical Sciences, College of Medicine, Taipei Medical University, Taipei 110, Taiwan; 3 Cell Physiology and Molecular Image Research Center, Wan Fang Hospital, Taipei Medical University, Taipei 116, Taiwan; 4 Drug Development and Value Creation Research Center, Kaohsiung Medical University, Kaohsiung 807, Taiwan

**Keywords:** c-Myc, PRMT1, olaparib, triple-negative breast cancer

## Abstract

Treatment of triple-negative breast cancer (TNBC) remains an unmet clinical need owing to its lack of an efficient therapeutic target. The targeting of DNA repair by poly(ADP-ribose) polymerase (PARP) inhibitors has shown benefit for patients with the BRCA variation. However, sensitivities to the PARP inhibitors were reported regardless of BRCA status. Thus, exploring the underlying mechanisms is imperative. Herein, we identified that breast cancer cells with an elevated expression of protein arginine methyl transferase 1 (PRMT1) was associated with therapeutic sensitivity to the PARP inhibitor olaparib. The results of cell viability and colony formation assays indicated that the suppression of PRMT1 by small hairpin RNA or by the chemical inhibitor increased sensitivity to olaparib in human TNBC MDA-MB-231 and BT549 cells. Bioinformatic analysis revealed that PRMT1 expression was significantly associated with the MYC signature, and TNBC cells with higher PRMT1 and the MYC signature were associated with therapeutic sensitivity to olaparib. Mechanistic studies further demonstrated that knockdown of PRMT1 reduced the c-Myc protein level and downregulated the expression of MYC downstream targets, whereas overexpression of PRMT1 enhanced c-Myc protein expression. Moreover, the overexpression of PRMT1 promoted c-Myc protein stability, and the inhibition of PRMT1 downregulated c-Myc protein stability. Accordingly, the knockdown of PRMT1 inhibited homologous recombination gene expression. These data indicate that PRMT1 is instrumental in regulating DNA repair, at least in part, by modulating c-Myc signaling. Our data highlighted the PRMT1/c-Myc network as a potential therapeutic target in patients with TNBC.

## 1. Introduction

Breast cancer is the most commonly diagnosed heterogeneous malignancy and the leading cause of cancer-related death in women worldwide [1]. Breast cancer is stratified into luminal, human epidermal receptor 2 (HER2)-enriched, and triple-negative breast cancer (TNBC) subtypes, according to the presence of distinct molecular markers, including the estrogen receptor (ER), progesterone receptor (PR), and HER2 [2]. Among these, TNBC characterized by the absence of the ER, PR, and HER2, accounts for approximately 10–20% of all breast cancer cases and is the most malignant subtype of breast cancer because of high heterogeneity, aggressiveness, and lack of treatment options [3,4]. Owing to a deficiency of efficient therapeutic targets and TNBC’s aggressive features, such as resistance to chemotherapy, higher invasiveness, and a stronger tendency to metastasize, patients with TNBC have poorer survival outcomes than those with non-TNBC [5,6]. Currently, the standard treatment for TNBC is chemotherapy. Unfortunately, patients with TNBC frequently develop resistance [7]. Thus, elucidating the genomic basis and molecular mechanism of chemoresistance in patients with TNBC to explore novel biomarkers is crucial for improving the accuracy of diagnosis and the efficacy of treatments for these patients.

Recently, targeting poly(ADP-ribose) polymerase (PARP) has shown therapeutic potential for patients with TNBC or ovarian cancer [8,9]. All PARP inhibitors impede PARP release on DNA to block DNA replication [10,11]. PARP inhibition blocks single-strand break repair and induces double-strand break (DSB) repair [12]. By contrast, cells with a deficiency in the homologous recombination (HR) mechanism, such as BRCA mutation, increase sensitivity to PARP inhibitors and lead to cell death [13]. However, approximately 40% of patients with the BRCA variations do not respond to PARP inhibitors or exhibit resistance. Some studies have reported that the therapeutic effects of PARP inhibitors occurred irrespective of BRCA status [14,15]. Thus, the underlying mechanism appears to be exceedingly complex and merits investigation.

In the search for novel biomarkers to predict the therapeutic efficacy of PARP inhibitors, we surveyed the Cancer Cell Line Encyclopedia (CCLE) in conjunction with the Genomics of Drug Sensitivity in Cancer (GDSC) database. We found that protein arginine methyl transferase 1 (PRMT1) was significantly correlated with therapeutic sensitivity to the PARP inhibitor olaparib in breast cancer cells. The PRMT family is responsible for protein methylation at the arginine residue [16]. In this family, PRMT1 is the most abundant PRMT and is responsible for 85% of protein methylation in mammalian cells [17,18]; moreover, it participates in regulating numerous cellular processes including signal transduction, epigenetic regulation, and double-strand break [19,20]. An aberrant expression of PRMT1 and other subtypes of PRMT has been identified in various malignancies [21,22]. In breast cancer, overexpression of PRMT1 promotes tumor metastasis by modulating EZH2 [23], and PRMT1 is associated with and prompts insulin-like growth factor I signaling in ER-positive breast cancer cells [24]. Moreover, PRMT1-mediated methylation of BRCA1 facilitates resistance to radiation therapy [25]. By contrast, methylation of CEBPα by PRMT1 impairs its tumor-suppressive function [26]. However, the molecular mechanism by which PRMT1 mediates resistance to PARP inhibitors in TNBC remains unclear.

The results presented herein demonstrate that breast cancer cells with elevated PRMT1 were closely associated with the therapeutic efficacy of olaparib. Gain-of-function and loss-of-function studies validated the crucial role of PRMT1 in TNBC cells’ sensitivity to olaparib. PRMT1 was significantly associated with MYC signature and regulated c-Myc protein stability; PRMT1 was also associated with HR gene expression modulation. Our findings delineate an alternative mechanism underpinning therapeutic resistance to PARP inhibitors through the PRMT1–MYC axis.

## 2. Materials and Methods

### 2.1. Cell Culture and Reagents

The human breast cancer cell lines MDA-MB-231 and BT549 were obtained from the American Type Culture Collection (ATCC, Manassas, VA, USA). Both cell lines were cultured in Dulbecco’s modified Eagle medium (DMEM), and the medium was supplemented with 7% fetal bovine serum (FBS; Corning, New York, NY, USA), 1% glutagro (Corning), and penicillin-streptomycin (Corning). Cells were cultured at 37 °C in a humidified atmosphere of 5% CO_2_. Olaparib was purchased from MedChemExpress, the PRMT1 inhibitor C7280948 was purchased from Sigma (St. Louis, MO, USA), and cycloheximide (CHX) was obtained from Sigma. pCMV3-C-FLAG-PRMT1 plasmid (HG11210-CF) was purchased from Sino Biological (Wu-Han, China), and pcDNA-cMyc-HA plasmid was kindly provided by Prof. Cheng Chia-Hsiung (Taipei Medical University). Antibodies against PRMT1 (GTX630187), c-Myc (GTX103436), and β-actin (GTX109639) were purchased from GeneTex (San Antonio, TX, USA).

### 2.2. Plasmids, Transfection, and Infection

Human PRMT1 shRNA#1 (TRCN0000290478) and shRNA#2 (TRCN0000290479) in pLKO.puro vectors were obtained from the National RNAi Core Facility (Academia Sinica, Taipei, Taiwan). Target sequences of shRNA#1 and shRNA#2 were 5′-CCGGCAGTACAAAGACTACAA-3′ and 5′-GTGTTCCAGTATCTCTGATTA-3′, respectively. Lentiviral preparation and viral infection were performed as previously described. In brief, 293T cells were cotransfected with pLKO.puro shRNA together with the pCMV-∆R8.91 and pMDG plasmids by using the PolyJet transfection reagent (SignaGen Laboratories, Ijamsville, MD, USA). Target cells were incubated with fresh complete DMEM mixed with virus-containing supernatants and polybrene (8 μg/mL) for another 24 h. Transduced cells were selected using puromycin (1–5 μg/mL) to generate stable cell lines. shRNA targeting LacZ was used as a negative control shRNA. For PRMT1 and cMyc overexpression, pCMV3-C-FLAG-PRMT1 plasmid and pcDNA3-cMyc-HA plasmid were transiently transfected into target cells by using the PolyJet transfection reagent. Efficiency of transfection was confirmed by using Western blot analysis after 48 h [27].

### 2.3. MTT Assay

Cell viability was estimated by using a 3-(4,5-dimethylthiazol-2-yl)-2,5-diphenyltetrazolium bromide solution (MTT) assay. MDA-MB-231 and BT549 cells were seeded at a density of 3 × 10^4^ cells/well in 24-well plates or 4 × 10^3^ cells/well in 48-well plates until they reached 50–60% confluence. Cells were refreshed with DMEM containing 3.5% FBS and were treated with olaparib or PRMT1 chemical inhibitor C7280948 at the indicated concentrations for 7 days. Fresh media containing drugs were replaced every 3 days. After treatments, cells were incubated with MTT (10 mg/mL) for 1 h. The MTT solution containing the medium was removed after incubation, and isopropanol was added to solubilize the formazan crystals for analysis. Cell viability was determined by detecting the absorbance at 570 nm and is expressed as the percentage of MTT reduction, assigning 100% to the value of the absorbance of control cells.

### 2.4. Colony Formation Assay

MDA-MB-231 and BT549 cells were seeded at a density of 1000 cells/well in 24-well plates. The next day, cells were refreshed with 3.5% FBS-containing DMEM and treated with olaparib or PRMT1 inhibitor C7280948 at the indicated concentrations for 10 days. Fresh media containing drugs were replaced every 3 days. After treatments, the cells were fixed with methanol for 30 min and stained with 0.5% crystal violet, and the plates with colonies were photographed.

### 2.5. Western Blotting

MDA-MB-231 and BT549 cells were lysed in ice-cold radioimmunoprecipitation assay buffer supplemented with a protease and phosphatase inhibitor cocktail (Roche, Mannheim, German). Cell lysates were subsequently analyzed by using Western blotting. Equal amounts of protein were separated using sodium dodecyl sulfate–polyacrylamide gel electrophoresis and transferred to polyvinylidene difluoride membranes (Millipore, Bedford, MA, USA). Membranes were blocked with 1% bovine serum albumin/Tris-buffered saline with Tween-20 (TBST) blocking buffer for 30 min and then incubated overnight at 4°C with specific primary antibodies. Membranes were washed three times with the TBST wash buffer, followed by incubation with a horseradish peroxidase–conjugated secondary antibody (Jackson ImmunoResearch, West Grove, PA, USA) at room temperature for 1 h. Bands were detected with an enhanced chemiluminescence system (Millipore). Western blotting was performed at least three times, and representative experiments are presented. Quantifications of protein levels were carried out by using Image J software v1.5.1, (U. S. National Institutes of Health, Bethesda, Maryland, USA).

### 2.6. Real-Time Polymerase Chain Reaction

RNA was extracted from MDA-MB-231 and BT549 cells by using a GENEzol TriRNA Pure kit (Geneaid, Taipei, Taiwan), and 1 μg of total RNA was reverse transcribed with Moloney murine leukemia virus reverse transcriptase (Promega, Madison, WI, USA). Complementary DNA was amplified with GoTaq qPCR Master Mix (Promega) in a StepOne Plus real-time polymerase chain reaction (PCR) system (Applied Biosystems, Darmstadt, Germany) with specific primers as follows: PRMT1, 5′-GAGGCCGCGAACTGCATCAT-3′ (sense) and 5′-TGGCTTTGACGATCTTCACC-3′ (antisense); E2F1, 5′-GGATTTCACACCTTTTCCTGGAT-3′ (sense) and 5′-CCTGGAAACTGACCATCAGTACCT-3′ (antisense); CDK4, 5′-GGACATATCTGGACAAGGCACC-3′ (sense) and 5′-ACTGTTCCACCACTTGTCACCAG-3′ (antisense); CCND1, 5′-CATCTACACCGACAACTCCATC-3′ (sense) and 5′-TCTGGCATTTTGGAGAGGAAG-3′ (antisense); β-catenin, 5′-TCTGAGGACAAGCCACAAGATTACA -3′ (sense) and 5′-TGGGCACCAATATCAAGTCCAA -3′ (antisense); LDHA, 5′-GATTCCAGTGTGCCTGTATGG -3′ (sense) and 5′-CTACAGAGAGTCCAATAGCCC-3′ (antisense); eIF4E, 5′-TGGCGACTGTCGAACCG-3′ (sense) and 5′-AGATTCCGTTTTCTCCTCTTCTGTAG-3′ (antisense); RAD51, 5′-GGTCTGGTGGTCTGTGTTGA-3′ (sense) and 5′-GGTGAAGGAAAGGCCATGTA-3′ (antisense); BRCA2, 5′-TGCCTGAAAACCAGATGACTATC-3′ (sense) and 5′-AGGCCAGCAAACTTCCGTTTA-3′ (antisense); RPA3, 5′-AAGCCTGTCTGCTTCGTAGGGA-3′ (sense) and 5′-CGGTTACTCTTCCAACCACTTCC-3′ (antisense); ATM, 5′-CAGGGTAGTTTAGTTGAGGTTGACAG-3′ (sense) and 5′-CTATACTGGTGGTCAGTGCCAAAGT-3′ (antisense); and β-actin, 5′-AAGTCCCTTGCCATCCTAAAA -3′ (sense) and 5′-ATGCTATCACCTCCCCTGTG-3′ (antisense). Results were calculated using the ΔΔCT equation and are expressed as multiples of change relative to a control sample.

### 2.7. Clinical and Molecular Data Acquisition

The raw gene expression data of PRMT1, MYC, and MYC-targeted genes, including *TERT*, *CDK4*, *ODC1*, *MDM2*, *TFAP4*, *E2F1*, *CCNE1*, and *CCNB1*, obtained by RNA sequencing along with clinical data were downloaded from The Cancer Genome Atlas (TCGA) (https://www.cbioportal.org/, accessed on 20 March 2021), CCLE (https://portals.broadinstitute.org/ccle, accessed on 10 May 2021), and Gene Expression Omnibus (accession nos. GSE18864 and GSE135565 [28,29]) databases. Units of mRNA levels were z-scores relative to all samples (log RNA Seq V2 RSEM), and units of mRNA in the CCLE were log2-normalized RNA expressions. Analyses of associations of PRMT1 with all hallmarks were performed using gene set enrichment analysis (GSEA) (https://www.gsea-msigdb.org, accessed on 17 June 2021). The gene lists of detected gene sets were obtained from the Molecular Signature Database (https://www.gsea-msigdb.org/gsea/msigdb, accessed on 17 June 2021). A high-expression group was defined as occurring in greater than 25% of patients [30]. Data of drug sensitivities in breast cancer cell lines were obtained from the GDSC database (https://www.cancerrxgene.org/, accessed on 10 May 2021). Cell groups susceptible and resistant to drugs were stratified according to optimal half-maximal inhibitory concentration (IC_50_) cutoff values [31].

### 2.8. Statistical Analyses

Data are presented as the mean ± standard deviation of three independent experiments. Statistical significance was determined by an unpaired, two-tailed Student’s *t* test unless stated otherwise (*p < 0.05; **p < 0.01; ***p < 0.001). Pearson’s correlation test was used to estimate the associations between mRNA levels of PRMT1, MYC, and MYC-targeted genes. Survival probabilities were evaluated using log-rank tests. All statistical analyses were performed. using GraphPad Prism 6.0 software.

## 3. Results

### 3.1. PRMT1 Expression Is Associated with Therapeutic Response to Olaparib in Breast Cancer Cells

**Figure 1:**
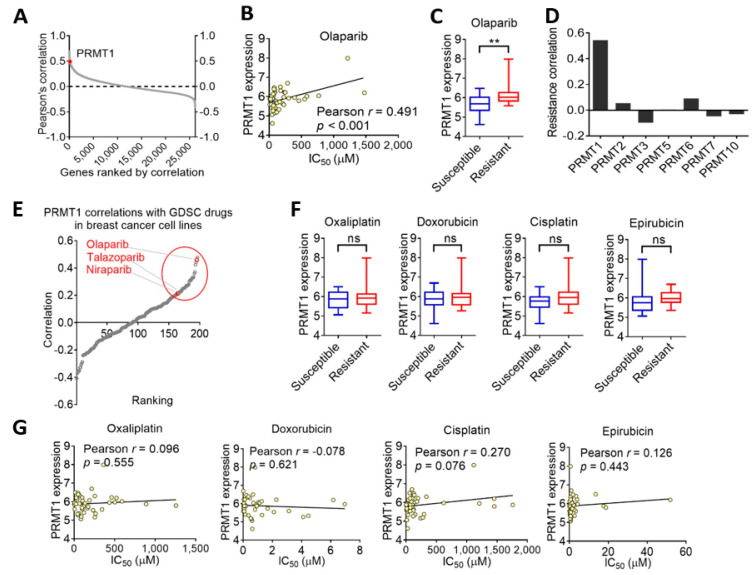
Breast cancer cells with elevated protein arginine methyl transferase 1 (PRMT1) expression levels are associated with therapeutic resistance to olaparib. (**A**) Correlations of sensitivities to olaparib with transcriptome-wide mRNA expression in breast cancer cell lines according to the Cancer Cell Line Encyclopedia (CCLE) datasets and the Genomics of Drug Sensitivity in Cancer (GDSC) database. PRMT1 is labeled with the red dot. (**B**) Box plot with Tukey’s test whiskers showing PRMT1 expression levels in olaparib-susceptible and -resistant breast cancer cell lines according to CCLE breast cancer dataset along with GDSC database. ** *p* < 0.01 was considered statistically significant and determined by using unpaired *t* tests. (**C)** Scatterplot of PRMT1 expression and half-maximal inhibitory concentration (IC_50_) of olaparib in breast cancer cell lines. Correlation coefficient was obtained by using Pearson’s test. Unit of PRMT1 mRNA was log2-normalized RNA expression. (**D**) Bar charts showing that PRMT1 has the strongest correlation with olaparib resistance among PRMT subtypes in breast cancer cell lines. (**E**) Correlations of PRMT1 and 200 drug sensitivities in breast cancer cell lines according to GDSC database and CCLE dataset. Red-labeled drugs are poly(ADP-ribose) polymerase inhibitors. (**F**) Box plots with PRMT1 expression levels among four conventional chemotherapy drugs (oxaliplatin, doxorubicin, cisplatin, and epirubicin) for drug-susceptible and -resistant breast cancer cell lines. ns, not significant. (**G**) Scatterplots of Pearson’s correlation analysis of PRMT1 and IC_50_ of four chemotherapy drugs in breast cancer cell lines.

To investigate potential genes associated with olaparib resistance, we explored the GDSC database and searched the IC_50_ value of olaparib and gene expression patterns in various breast cancer cell lines from the CCLE datasets. Intriguingly, the correlations of olaparib sensitivities with transcriptome-wide mRNA expression in breast cancer cell lines revealed that the *PRMT1* gene was within the top ranks (Figure 1A), and breast cancer cells with an elevated expression of PRMT1 were associated with therapeutic resistance to olaparib (Figure 1B). Pearson’s correlation indicated that PRMT1 was significantly correlated with olaparib sensitivity in 44 breast cancer cell lines (*r* = 0.491, *p* < 0.001; Figure 1C). We also investigated the PRMT family and identified that olaparib sensitivity was exclusively associated with PRMT1 but not with other PRMT subtypes (Figure 1D). We further analyzed the correlations between PRMT1 and sensitivities to 200 anticancer drugs in breast cancer cells (Figure 1E). Notably, PRMT1 was closely associated with therapeutic sensitivity to PARP inhibitors, including olaparib, talazoparib, and niraparib (Figure 1E) but not with other conventional chemotherapy drugs such as oxaliplatin (*r* = 0.096), doxorubicin (*r* = −0.078), cisplatin (*r* = 0.270), or epirubicin (*r* = 0.126; Figure 1F,G), suggesting that PRMT1 may be a reliable marker to predict a therapeutic outcome with PARP inhibitors.

### 3.2. Inhibition of PRMT1 Increases Sensitivity to Olaparib in TNBC Cells

**Figure 2:**
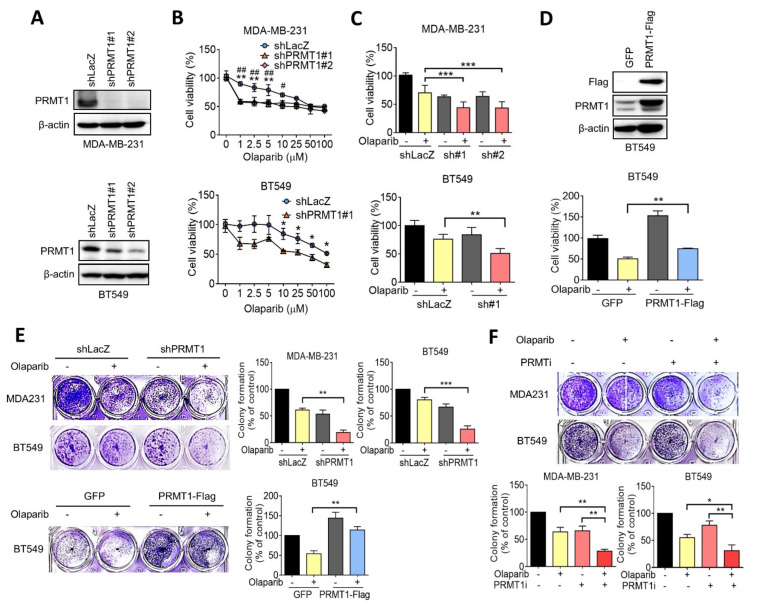
PRMT1 correlates with olaparib resistance in triple-negative breast cancer (TNBC) cells. (**A**) Western blotting indicating knockdown efficacies of PRMT1 shRNAs confirmed by MDA-MB-231 and BT549 cells. β-actin was used as an internal control. (**B**) MDA-MB-231 and BT549 cells were treated with various doses of olaparib (0, 1, 2.5, 5, 10, 25, 50, and 100 μM) for 7 days. Fresh medium with olaparib was replaced every 3 days. Cell viability was measured by using 3-(4,5-dimethylthiazol-2-yl)-2,5-diphenyltetrazolium bromide solution (MTT) assay. Data are expressed as a percentage of the control. Values are expressed as the mean ± standard error. ** *p* < 0.01 (shLacZ compared to shPRMT1#1); ## *p* < 0.01 and # *p* < 0.05 (shLacZ compared to shPRMT1#2). (**C**) MDA-MB-231 and BT549 were respectively treated with 5 μM and 10 μM olaparib for 7 days. An MTT assay was used to evaluate cell viability. (**D**) Western blotting indicating the efficacy of PRMT1 overexpression confirmed by BT549 cells. PRMT1 vector was labeled with a Flag tag, and BT549 cells were transiently transfected with PRMT1-Flag plasmid (upper panel). BT549 cells were treated with 10 μM olaparib for 7 days as previously described, and an MTT assay was performed. (**E**) Representative images of colony formation for MDA-MB-231 and BT549 treated with olaparib (5 μM and 10 μM) for 10 days. Fresh medium containing olaparib was replaced every 3 days. (**F**) Colony formation for MDA-MB-231 and BT549 cells treated with olaparib (5 μM and 10 μM) combined with PRMT1 inhibitor C7280948 (80 μM) for 10 days. Quantification of clonogenic formation was carried out using Image J software. Data were obtained from three biological replicates. Unpaired *t* tests were performed to compare expression levels in two groups. * *p* < 0.05, **p < 0.01, *** *p* < 0.001 were considered statistically significant.

To clarify the role of PRMT1 in the olaparib sensitivity of TNBC, short hairpin RNAs against PRMT1 were made to knocked down expression, and we confirmed the knockdown efficacy in MDA-MB-231 and BT549 cells (Figure 2A). MDA-MB-231 and BT549 cells were subsequently treated with various concentrations of olaparib for 7 days, and cell viability was determined using an MTT assay. The results revealed that the suppression of PRMT1 significantly reduced cell viability in response to olaparib treatment in both MDA-MB-231 and BT549 cells compared with that in control knockdown cells (Figure 2B,C). The IC_50_ values of olaparib in control was 80.9 μM, and those in PRMT1 knockdown MDA-MB-231 cells were 51.4 μM and 15.0 μM, respectively. The IC_50_ values of olaparib in control and PRMT1-silencing BT549 cells were 146.5 μM and 18.4 μM, respectively (Figure 2B). By contrast, the growth inhibition by olaparib was rescued by ectopic expression of PRMT1 in BT549 cells (Figure 2D). Similarly, the colony formation assay indicated that knockdown of PRMT1 substantially reduced cell growth in response to olaparib (Figure 2E), whereas the ectopic overexpression of PRMT1 recapitulated the growth inhibition by olaparib (Figure 2E). Similarly, the combination of olaparib with the chemical inhibitor of PRMT1 enhanced growth inhibition in both MDA-MB-231 and BT549 cells, as determined by a colony formation assay (Figure 2F). These data suggest that PRMT1 expression plays a crucial role in olaparib sensitivity in TNBC cells.

### 3.3. PRMT1 Expression Is Associated with MYC Signature

**Figure 3:**
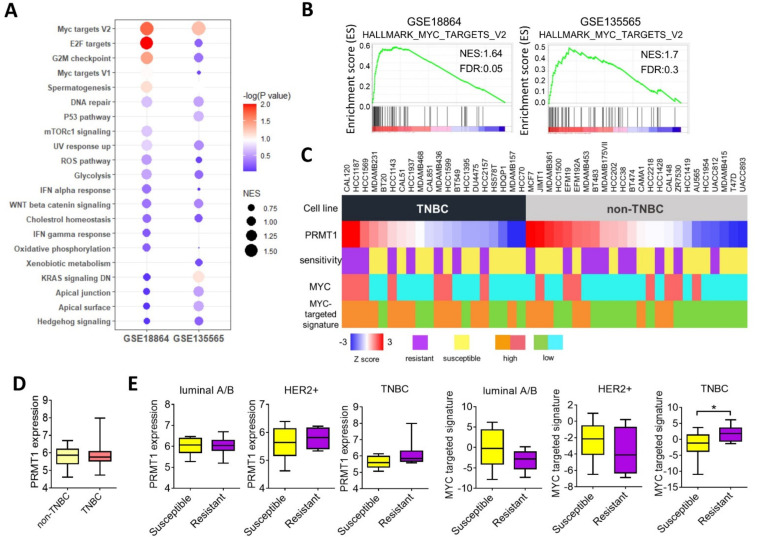
MYC targets are associated with PRMT1 and olaparib resistance in TNBC. (**A**) Bubble plot based on gene set enrichment analysis (GSEA) showing enrichment of PRMT1 with cancer hallmarks in the GSE18864 and GSE135565 datasets. Size of bubbles was ranked by normalized enrichment score, and bubble color was determined by *p* value. (**B**) Representative GSEA plots revealing associations between PRMT1 expression and MYC targets in the GSE18864 and GSE135565 datasets. (**C**) Schematic of PRMT1 expression, olaparib sensitivity, MYC expression, and MYC-targeted signature in breast cancer cell lines categorized into TNBC and non-TNBC groups using the Genomics of Drug Sensitivity in Cancer database (GDSC) and the Cancer Cell Line Encyclopedia (CCLE) dataset. The olaparib-resistant group was defined as half-maximal inhibitory concentration greater than 150 μM. The MYC high-expressing group was defined as MYC expression higher than average. The MYC-targeted signature was evaluated by combination score of eight MYC-targeted genes, and a score higher than average was classified into the high-expressing group. (**D**) Box plot with PRMT1 expression levels in TNBC and non-TNBC cell lines. (**E**) Box plots indicating PRMT1 expression and MYC-targeted signature in olaparib-susceptible and -resistant groups in breast cancer cell lines classified as breast cancer subtypes. Olaparib-susceptible and -resistant groups were defined as previously described. * *p* < 0.05 was considered statistically significant and determined by using unpaired *t* tests.

**Figure 4:**
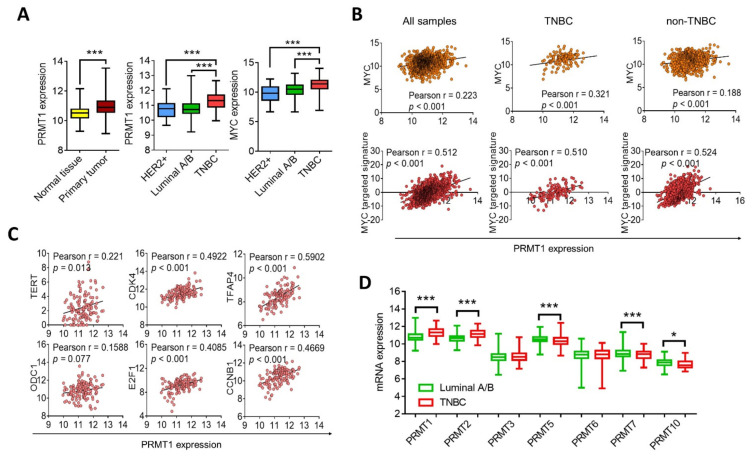
PRMT1 expression level is associated with MYC signature in TNBC. (**A**) Box plots with expression levels of PRMT1 and MYC among breast cancer subtypes using the TCGA_BRCA dataset. Patients with breast cancer were characterized by molecular subtypes, including luminal A/B (PRMT1: *n* = 574; MYC: *n* = 508), human epidermal receptor 2 (HER2) (PRMT1: *n* = 31; MYC: *n* = 30), TNBC (PRMT1: *n* = 123; MYC: *n* = 123), and normal adjacent tissues (PRMT1: *n* = 114; ***p < 0.001 was considered statistically significant and determined by using unpaired *t* tests). Units of mRNA levels were z-scores relative to all samples (log RNA Seq V2 RSEM). (**B**) Scatterplots showing Pearson’s correlation analysis of PRMT1 with MYC (upper panel) and MYC-targeted signature (lower panel) in TCGA_BRCA cohorts. (**C**) Scatterplots of PRMT1 expression and six MYC-targeted gene expression levels respectively in patients with TNBC (*n* = 125) using TCGA_BRCA cohorts. Correlation coefficients were calculated by using Pearson’s test. (**D**) Box plots showing expression levels of PRMT subtypes in patients with luminal A/B and with TNBC using TCGA_BRCA datasets. Unpaired *t* tests were performed to compare expression levels in two groups. * *p* < 0.05, *** *p* < 0.001 were considered statistically significant.

To clarify the molecular function of PRMT1 in mediating olaparib sensitivity, we analyzed associations between PRMT1 and the cancer hallmarks by using GSEA. The results demonstrated that the c-Myc and E2F target signatures had the most significant correlation with PRMT1 in two TNBC cohorts (Figure 3A,B). We further analyzed the mRNA levels of PRMT1 and c-Myc regulatory genes and their associations with olaparib sensitivity in breast cancer cells by using the CCLE database (Figure 3C). The results revealed that PRMT1 expression was not significantly upregulated in TNBC cells compared with non-TNBC cells (Figure 3D). However, breast cancer cells that exhibited resistance to olaparib were closely associated with PRMT1 or the MYC-targeted signature, specifically in TNBC cell lines (Figure 3E). These data support the notion of PRMT1 as a reliable therapeutic target for olaparib treatment in TNBC. Subsequent analysis of the transcriptome from the breast cancer cohort in TCGA_BRCA indicated that PRMT1 was elevated in breast tumor tissues compared with normal tissues (Figure 4A). In addition, PRMT1 was overexpressed in basal-like TNBC compared with HER2 or luminal subtypes (Figure 4A). Similarly, patients with TNBC exhibited elevated expression of c-Myc compared with those with non-TNBC (Figure 4A). Moreover, PRMT1 was significantly associated with c-Myc and c-Myc-targeted genes (Figure 4B), and notably, the correlation coefficients between PRMT1 with c-Myc or the MYC-targeted signature were more significant in patients with TNBC than in those with non-TNBC (Figure 4B). Consistently, PRMT1 expression was positively correlated with the MYC downstream genes (Figure 4C). We also analyzed the expression patterns of PRMT subtypes in patients with breast cancer, and the results validated that PRMT1, PRMT2 and PRMT4 were elevated in patients with TNBC compared with levels in those with non-TNBC (Figure 4D). By contrast, expression levels of PRMT5, PRMT7, and PRMT10 were downregulated in patients with TNBC (Figure 4D). These data indicate that the PRMT1–MYC axis may be instrumental in TNBC.

### 3.4. PRMT1 Regulates c-Myc Protein Stability

**Figure 5:**
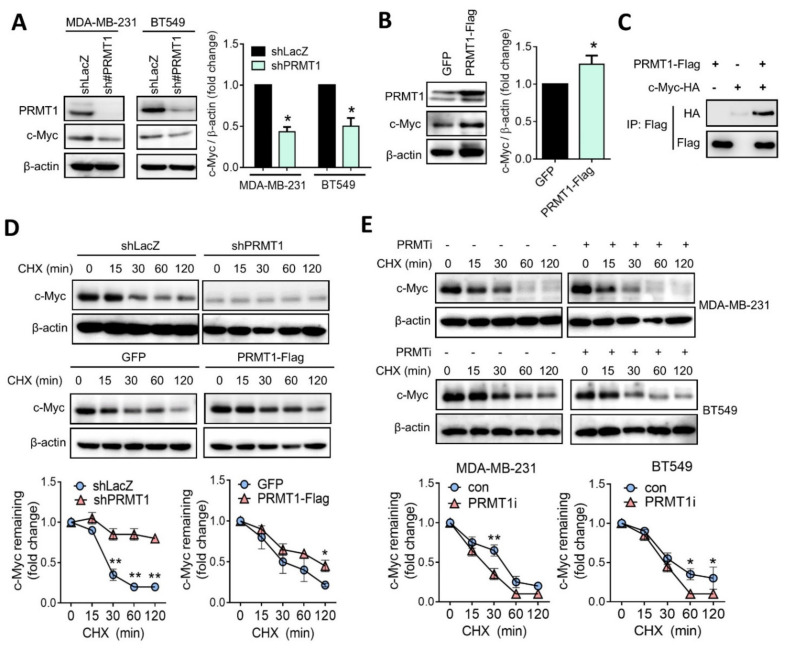
PRMT1 mediates c-Myc protein level by regulating its stability. (**A**) Protein levels of c-Myc were analyzed using Western blotting in PRMT1-knockdown MDA-MB-231 and BT549 cells. Relative fold changes of c-Myc to β-actin protein level was shown (right panel). (**B**) PRMT1 and c-Myc protein levels analyzed using by Western blotting in PRMT1-overexpressing BT549 cells. GFP, green fluorescent protein. BT549 cells were transiently transfected with PRMT1-Flag plasmid, and protein lysates were subjected to Western blot analysis. Relative fold changes of c-Myc to β-actin protein level was shown (right panel). (**C**) Co-immunoprecipitation analysis of PRMT1-Flag and c-Myc-HA in 293T cells. (**D**) Western blot analysis of c-Myc protein level in shLacZ and shPRMT1 BT549 cells treated with CHX (50 μM) for the indicated times (0, 15, 30, 60, and 120 min). (Upper panel). Western blot analysis of c-Myc protein levels in GFP and PRMT1-overexpressing BT549 cells treated with CHX (50 μM) for the indicated times (0, 15, 30, 60, and 120 min). (Lower panel). Relative fold changes of c-Myc to β-actin protein levels were shown. (**E**) c-Myc protein levels were detected using by Western blotting in MDA-MB-231 and BT549 cells following treatment with CHX (50 μM) and PRMT1 inhibitor (40 μM) or CHX (50 μM) alone for the indicated times (0, 15, 30, 60, and 120 min). Western blots were performed from three biological replicates, and quantifications of protein levels were carried out by using Image J software. Unpaired *t* tests were performed to compare expression levels in two groups. * *p* < 0.05, ** *p* < 0.01 were considered statistically significant.

To validate the regulatory mechanism between PRMT1 and c-Myc, we examined the protein level of c-Myc with or without PRMT1 expression. Western blot analysis revealed that the suppression of PRMT1 substantially downregulated the c-Myc protein level in MDA-MB-231 and BT549 cells (Figure 5A). By contrast, the ectopic expression of PRMT1 upregulated the c-Myc protein level in BT549 cells (Figure 5B). Moreover, a co-immunoprecipitation assay revealed that PRMT1 associated with c-Myc (Figure 5C). To examine the effect of PRMT1 on c-Myc protein stability, cells were treated with a protein synthesis inhibitor, cycloheximide (CHX). However, the c-Myc protein level did not obviously decline in PRMT1-knockdown cells when exposed to CHX (Figure 5D). We speculated that the basal protein level of c-Myc was relatively low in PRMT1 stable knockdown cells. In support of this notion, BT549 cells expressed ectopically were ectopic expression with PRMT1, and the results showed that the overexpression of PRMT1 increased c-Myc protein stability in the presence of CHX (Figure 5D). Moreover, the inhibition of PRMT1 by the chemical inhibitor reduced the c-Myc protein level. The inhibition of PRMT1 resulted in the shortening of the c-Myc protein half-life from 1 h to 30 min (Figure 5E), suggesting that PRMT1 may regulate c-Myc protein stability. These findings indicate that PRMT1 associates with and stabilizes c-Myc protein.

### 3.5. Suppression of PRMT1 Downregulates HR Gene Expression

**Figure 6:**
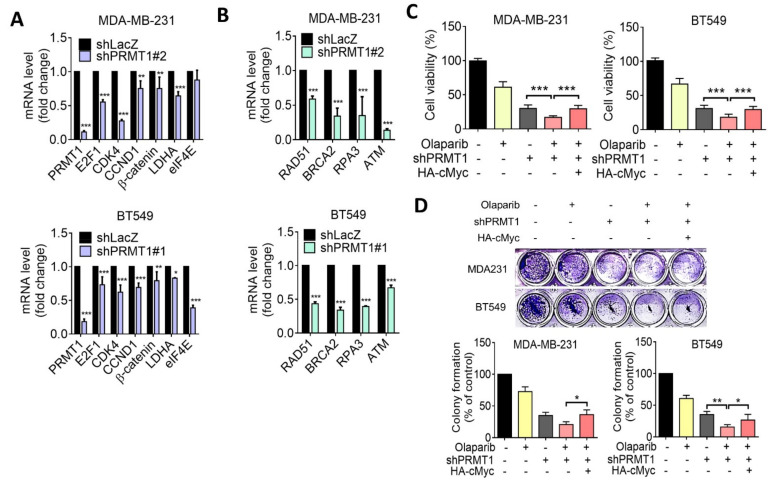
PRMT1 regulates c-Myc downstream genes and homologous recombination (HR) genes in TNBC cells. (**A**) Reverse transcriptase quantitative polymerase chain reaction (RT-qPCR) analysis of MYC-targeted gene expression levels in PRMT1-knockdown MDA-MB-231 and BT549 cells. Knockdown efficacies of PRMT1 shRNAs in MDA-MB-231 and BT549 cells are shown. (**B**) RT-qPCR analysis of HR gene expression levels in PRMT1-knockdown MDA-MB-231 and BT549 cells. (**C** and **D**) MTT assay and colony formation assay showing that c-Myc overexpression rescued PRMT1 silencing–mediated growth inhibition in response to olaparib in MDA-MB-231 and BT549 cells. Quantification of clonogenic formation was carried out using Image J software. Data were obtained from three biological replicates. Unpaired *t* tests were performed to compare expression levels in two groups. * *p* < 0.05, ** *p* < 0.01, *** *p* < 0.001 were considered statistically significant.

Homologous recombination (HR) plays essential roles in the repair of DNA double strand break (DSB), and previous studies have identified that c-Myc regulates several DSB genes [32]. Moreover, deficiency in HR resulted in enhancing cell death triggered by PARP inhibitors [33]. We subsequently performed reverse transcriptase quantitative PCR (RT-qPCR) analysis to assess regulation of HR genes by PRMT1. The RT-qPCR analysis revealed that knockdown of PRMT1 downregulated c-Myc downstream targets (Figure 6A). Accordingly, knockdown of PRMT1 significantly downregulated HR-related gene expression in MDA-MB-231 and BT549 cells (Figure 6B). To further examine the participation of c-Myc in PRMT1-mediated drug sensitivity, cells were ectopically expressed with c-Myc. The results of MTT and colony formation assays indicated that overexpression of c-Myc partially restored PRMT1 silencing–mediated growth inhibition in response to olaparib (Figure 6C,D). These data suggest that c-Myc–regulated HR may contribute to the resistance to olaparib induced by PRMT1.

## 4. Discussion

The inhibition of PARP exhibits a therapeutic benefit for patients with cancer with the BRCA variation, especially in patients with TNBC and those with ovarian cancer [34]. However, several studies have reported that therapeutic efficacy of PARP inhibitors was independent of BRCA status [14], indicating that the underlying mechanism is exceedingly complex and warrants investigation. Herein, we identified that PRMT1 was responsible for olaparib sensitivity in TNBC cells. PRMT1 was elevated in breast cancer cells, and PRMT1 was overexpressed in patients with TNBC. The expression of PRMT1 was significantly associated with the MYC signature. We also identified that PRMT1 regulated c-Myc protein stability, and suppression of PRMT1 increased sensitivity to olaparib, at least in part, by c-Myc-mediated HR gene expression. The combination of olaparib and a PRMT1 inhibitor enhanced growth inhibition in TNBC cells. However, we found that suppression of PRMT1 did not significantly increase sensitivity towards doxorubicin (Supplemental Figure S1). These data were similar to the results from *in silico* analyses. In our study, we found that PRMT1 regulates c-Myc and modulates HR genes expression. DNA repair mechanism is critical for the resistance to genotoxic agents such as cisplatin, therefore, PRMT1 expression may have a role in chemoresistance under different contexts [35]. Nevertheless, our study pinpointed an alternative mechanism that was mediated by the PRMT1-MYC axis in the sensitivity to olaparib. This data demonstrated that inhibition of PRMT1 showed more susceptibility towards the PARP inhibitors, which suggests its potential as a therapeutic target in patients with TNBC.

Protein methylation by members of the PRMT family regulates numerous cellular responses. PRMT1 is the most abundant PRMT and is overexpressed in diverse malignancies [36]. The methylation of heat shock protein 70 by PRMT1 stabilized BCL2 mRNA and led to pancreatic cancer resistance to therapeutics [37]. PRMT1 promoted tumor-initiating capability in esophageal squamous cell carcinoma through histone arginine methylation [36]. Moreover, PRMT1 methylated an epidermal growth factor (EGF) receptor to regulate EGF signaling and confer resistance to cetuximab [38]. These data suggest a crucial role of PRMT1 in mediating therapeutic resistance by modulating various signaling molecules. Nevertheless, the role of PRMT1 in olaparib resistance to TNBC cells remains unclear. By using *in silico* data analysis, we found that PRMT1 was exclusively associated with therapeutic response to the PARP inhibitors. We further validated that PRMT1 plays a critical role in treatment response to olaparib. Inhibition of PRMT1 by shRNA or a chemical inhibitor sensitized TNBC cells to olaparib. Mechanistically, PRMT1 enhanced c-Myc protein stability and regulated HR-related gene expression. Moreover, patients with TNBC overexpressed PRMT1 and c-Myc, and a significant association was observed between PRMT1 and the MYC signature.

The oncoprotein MYC controls the expression of DNA DSB repair genes involved in nonhomologous end-joining and HR. The inhibition of MYC sensitized cancer cells to DNA damage [39,40,41,42]. Ning et al. recently reported that Myc promoted HR to mediate resistance to PARP inhibitors in glioma [43]. The inhibition of MYC binding protein impaired HR gene expression and led to an increase in the sensitivity of TNBC cells to olaparib. Accordingly, the association of MYC and the PRMT family has previously been reported. Chaturvedi et al. identified that PRMT5 interacted with and stabilized MYC in glioma [44], and Annarita et al. proposed that PRMT1 methylated c-Myc in the presence of PRMT5 in glioma stem cells [45]. In our study, we demonstrated that PRMT1 associated with and stabilized c-Myc protein, whereas suppression of PRMT1 reduced the c-Myc protein stability. Notably, we found that c-Myc protein stability was decreased by treatment with the inhibitor of PRMT1; however, c-Myc protein level was relatively low and showed less response in PRMT1-knockdown cells. In addition, we found that patients with TNBC overexpressed PRMT1 but not PRMT5. These data may suggest the crucial role of PRMT1 in modulating c-Myc stability, thereby bolstering therapeutic sensitivity to PARP inhibitors for TNBC. Notably, inhibition of PRMT1 by the chemical inhibitor downregulated c-Myc and sensitized TNBC cells to olaparib, suggesting that the methylation activity of PRMT1 may have a role in mediating resistance to PARP inhibitors. A previous study reported that arginine methylation of c-Myc by PRMT1 facilitated acetyltransferase p300 binding and c-Myc transcriptional activation in macrophages [46]. Therefore, it is worth to further investigating the role of c-Myc methylation in the resistance to olaparib in TNBC, and the importance of the PRMT1–MYC network should be investigated in terms of resistance and a suitable therapeutic approach.

Taken together, the PRMT1–MYC signaling axis confers therapeutic resistance to olaparib. The overexpression of PRMT1 is significantly associated with the MYC signature in TNBC. Targeting PRMT1 enhances the efficacy of olaparib, shedding light on countering drug resistance. PRMT1 thus shows promise as a target in the treatment of patients with TNBC.

## Data Availability

Not applicable.

## Supplementary Materials

The following are available online at https://www.mdpi.com/article/10.3390/jpm11101009/s1, Figure S1: Cell viability of BT549 cells in response to doxorubicin.

## References

[1] Baselga J., Norton L. (2002). Focus on breast cancer. Cancer Cell.

[2] Gupta G.K., Collier A.L., Lee D., Hoefer R.A., Zheleva V., Siewertsz van Reesema L.L., Tang-Tan A.M., Guye M.L., Chang D.Z., Winston J.S. (2020). Perspectives on Triple-Negative Breast Cancer: Current Treatment Strategies, Unmet Needs, and Potential Targets for Future Therapies. Cancers.

[3] Won K.A., Spruck C. (2020). Triple-negative breast cancer therapy: Current and future perspectives (Review). Int J. Oncol..

[4] Yin H., Xiong G., Guo S., Xu C., Xu R., Guo P., Shu D. (2019). Delivery of Anti-miRNA for Triple-Negative Breast Cancer Therapy Using RNA Nanoparticles Targeting Stem Cell Marker CD133. Mol. Ther..

[5] Bianchini G., Balko J.M., Mayer I.A., Sanders M.E., Gianni L. (2016). Triple-negative breast cancer: Challenges and opportunities of a heterogeneous disease. Nat. Rev. Clin. Oncol..

[6] Carey L., Winer E., Viale G., Cameron D., Gianni L. (2010). Triple-negative breast cancer: Disease entity or title of convenience?. Nat. Rev. Clin. Oncol..

[7] Garrido-Castro A.C., Lin N.U., Polyak K. (2019). Insights into Molecular Classifications of Triple-Negative Breast Cancer: Improving Patient Selection for Treatment. Cancer Discov..

[8] Sachdev E., Tabatabai R., Roy V., Rimel B.J., Mita M.M. (2019). PARP Inhibition in Cancer: An Update on Clinical Development. Target. Oncol..

[9] Mittica G., Ghisoni E., Giannone G., Genta S., Aglietta M., Sapino A., Valabrega G. (2018). PARP Inhibitors in Ovarian Cancer. Recent Pat. Anticancer Drug Discov..

[10] Slade D. (2020). PARP and PARG inhibitors in cancer treatment. Genes Dev..

[11] Han Y., Yu X., Li S., Tian Y., Liu C. (2020). New Perspectives for Resistance to PARP Inhibitors in Triple-Negative Breast Cancer. Front. Oncol..

[12] Curtin N.J., Szabo C. (2013). Therapeutic applications of PARP inhibitors: Anticancer therapy and beyond. Mol. Asp. Med..

[13] Javle M., Curtin N.J. (2011). The role of PARP in DNA repair and its therapeutic exploitation. Br. J. Cancer.

[14] Keung M.Y., Wu Y., Badar F., Vadgama J.V. (2020). Response of Breast Cancer Cells to PARP Inhibitors Is Independent of BRCA Status. J. Clin. Med..

[15] Faraoni I., Graziani G. (2018). Role of BRCA Mutations in Cancer Treatment with Poly(ADP-ribose) Polymerase (PARP) Inhibitors. Cancers.

[16] Qualmann B., Kessels M.M. (2021). The Role of Protein Arginine Methylation as Post-Translational Modification on Actin Cytoskeletal Components in Neuronal Structure and Function. Cells.

[17] Zhang X.P., Jiang Y.B., Zhong C.Q., Ma N., Zhang E.B., Zhang F., Li J.J., Deng Y.Z., Wang K., Xie D. (2018). PRMT1 Promoted HCC Growth and Metastasis In Vitro and In Vivo via Activating the STAT3 Signalling Pathway. Cell Physiol. Biochem..

[18] Hsu J.H., Hubbell-Engler B., Adelmant G., Huang J., Joyce C.E., Vazquez F., Weir B.A., Montgomery P., Tsherniak A., Giacomelli A.O. (2017). PRMT1-Mediated Translation Regulation Is a Crucial Vulnerability of Cancer. Cancer Res..

[19] Malbeteau L., Poulard C., Languilaire C., Mikaelian I., Flamant F., Le Romancer M., Corbo L. (2020). PRMT1 Is Critical for the Transcriptional Activity and the Stability of the Progesterone Receptor. iScience.

[20] Wang Y., Li X., Ge J., Liu M., Pang X., Liu J., Luo C., Xu Y., Zhao Q. (2021). The methyltransferase PRMT1 regulates γ-globin translation. J. Biol. Chem..

[21] Yang Y., Bedford M.T. (2013). Protein arginine methyltransferases and cancer. Nat. Rev. Cancer.

[22] Hua Z.-Y., Hansen J.N., He M., Dai S.-K., Choi Y., Fulton M.D., Lloyd S.M., Szemes M., Sen J., Ding H.-F. (2020). PRMT1 promotes neuroblastoma cell survival through ATF5. Oncogenesis.

[23] Choi H.J., Jhe Y.L., Kim J., Lim J.Y., Lee J.E., Shin M.K., Cheong J.H. (2020). FoxM1-dependent and fatty acid oxidation-mediated ROS modulation is a cell-intrinsic drug resistance mechanism in cancer stem-like cells. Redox Biol..

[24] Choucair A., Pham T.H., Omarjee S., Jacquemetton J., Kassem L., Trédan O., Rambaud J., Marangoni E., Corbo L., Treilleux I. (2019). The arginine methyltransferase PRMT1 regulates IGF-1 signaling in breast cancer. Oncogene.

[25] Montenegro M., González Guerrero R., Campo L., Piñero A., Cabezas-Herrera J., Rodríguez-López J. (2020). PRMT1-dependent methylation of BRCA1 contributes to the epigenetic defense of breast cancer cells against ionizing radiation. Sci. Rep..

[26] Liu L.M., Sun W.Z., Fan X.Z., Xu Y.L., Cheng M.B., Zhang Y. (2019). Methylation of C/EBPα by PRMT1 Inhibits Its Tumor-Suppressive Function in Breast Cancer. Cancer Res..

[27] Lee K.Y., Kuo T.C., Chou C.M., Hsu W.J., Lee W.C., Dai J.Z., Wu S.M., Lin C.W. (2020). Upregulation of CD109 Promotes the Epithelial-to-Mesenchymal Transition and Stemness Properties of Lung Adenocarcinomas via Activation of the Hippo-YAP Signaling. Cells.

[28] Buechler S.A., Gokmen-Polar Y., Badve S.S. (2016). RespondR signature to predict potential alternative therapies for taxane resistant triple-negative breast cancer patients. J. Clin. Oncol..

[29] Wang S., Zou X., Chen Y., Cho W.C., Zhou X. (2021). Effect of N6-Methyladenosine Regulators on Progression and Prognosis of Triple-Negative Breast Cancer. Front. Genet..

[30] Lai Y.-W., Hsu W.-J., Lee W.-Y., Chen C.-H., Tsai Y.-H., Dai J.-Z., Yang C.-C., Lin C.-W. (2021). Prognostic Value of a Glycolytic Signature and Its Regulation by Y-Box-Binding Protein 1 in Triple-Negative Breast Cancer. Cells.

[31] Hamard P.J., Santiago G.E., Liu F., Karl D.L., Martinez C., Man N., Mookhtiar A.K., Duffort S., Greenblatt S., Verdun R.E. (2018). PRMT5 Regulates DNA Repair by Controlling the Alternative Splicing of Histone-Modifying Enzymes. Cell Rep..

[32] Luoto K.R., Meng A.X., Wasylishen A.R., Zhao H., Coackley C.L., Penn L.Z., Bristow R.G. (2010). Tumor cell kill by c-MYC depletion: Role of MYC-regulated genes that control DNA double-strand break repair. Cancer Res..

[33] Helleday T., Bryant H.E., Schultz N. (2005). Poly(ADP-ribose) polymerase (PARP-1) in homologous recombination and as a target for cancer therapy. Cell Cycle.

[34] Pilié P.G., Gay C.M., Byers L.A., O’Connor M.J., Yap T.A. (2019). PARP Inhibitors: Extending Benefit Beyond *BRCA*-Mutant Cancers. Clin. Cancer Res..

[35] Musiani D., Giambruno R., Massignani E., Ippolito M.R., Maniaci M., Jammula S., Manganaro D., Cuomo A., Nicosia L., Pasini D. (2020). PRMT1 Is Recruited via DNA-PK to Chromatin Where It Sustains the Senescence-Associated Secretory Phenotype in Response to Cisplatin. Cell Rep..

[36] Zhao Y., Lu Q., Li C., Wang X., Jiang L., Huang L., Wang C., Chen H. (2019). PRMT1 regulates the tumour-initiating properties of esophageal squamous cell carcinoma through histone H4 arginine methylation coupled with transcriptional activation. Cell Death Dis..

[37] Wang L., Jia Z., Xie D., Zhao T., Tan Z., Zhang S., Kong F., Wei D., Xie K. (2020). Methylation of HSP70 Orchestrates Its Binding to and Stabilization of BCL2 mRNA and Renders Pancreatic Cancer Cells Resistant to Therapeutics. Cancer Res..

[38] Liao H.W., Hsu J.M., Xia W., Wang H.L., Wang Y.N., Chang W.C., Arold S.T., Chou C.K., Tsou P.H., Yamaguchi H. (2015). PRMT1-mediated methylation of the EGF receptor regulates signaling and cetuximab response. J. Clin. Investig..

[39] Li Z., Owonikoko T.K., Sun S.-Y., Ramalingam S.S., Doetsch P.W., Xiao Z.-Q., Khuri F.R., Curran W.J., Deng X. (2012). c-Myc suppression of DNA double-strand break repair. Neoplasia.

[40] Yi J., Liu C., Tao Z., Wang M., Jia Y., Sang X., Shen L., Xue Y., Jiang K., Luo F. (2019). MYC status as a determinant of synergistic response to Olaparib and Palbociclib in ovarian cancer. EBioMedicine.

[41] Campaner S., Amati B. (2012). Two sides of the Myc-induced DNA damage response: From tumor suppression to tumor maintenance. Cell Div..

[42] Vafa O., Wade M., Kern S., Beeche M., Pandita T.K., Hampton G.M., Wahl G.M. (2002). c-Myc can induce DNA damage, increase reactive oxygen species, and mitigate p53 function: A mechanism for oncogene-induced genetic instability. Mol. Cell.

[43] Ning J.-F., Stanciu M., Humphrey M.R., Gorham J., Wakimoto H., Nishihara R., Lees J., Zou L., Martuza R.L., Wakimoto H. (2019). Myc targeted CDK18 promotes ATR and homologous recombination to mediate PARP inhibitor resistance in glioblastoma. Nat. Commun..

[44] Chaturvedi N.K., Mahapatra S., Kesherwani V., Kling M.J., Shukla M., Ray S., Kanchan R., Perumal N., McGuire T.R., Sharp J.G. (2019). Role of protein arginine methyltransferase 5 in group 3 (MYC-driven) Medulloblastoma. BMC Cancer.

[45] Favia A., Salvatori L., Nanni S., Iwamoto-Stohl L.K., Valente S., Mai A., Scagnoli F., Fontanella R.A., Totta P., Nasi S. (2019). The Protein Arginine Methyltransferases 1 and 5 affect Myc properties in glioblastoma stem cells. Sci. Rep..

[46] Tikhanovich I., Zhao J., Bridges B., Kumer S., Roberts B., Weinman S.A. (2017). Arginine methylation regulates c-Myc-dependent transcription by altering promoter recruitment of the acetyltransferase p300. J. Biol. Chem..

