# Supplementary material for: PRMT1 Confers Resistance to Olaparib via Modulating MYC Signaling in Triple-Negative Breast Cancer
Source: J Pers Med. 2021 Oct 8;11(10):1009. doi: 10.3390/jpm11101009 (PMC8539542; doi:10.3390/jpm11101009)
Supplement: Supplementary file 1 [file jpm-11-01009-s001.zip › jpm-1379853-supplementary.pdf]

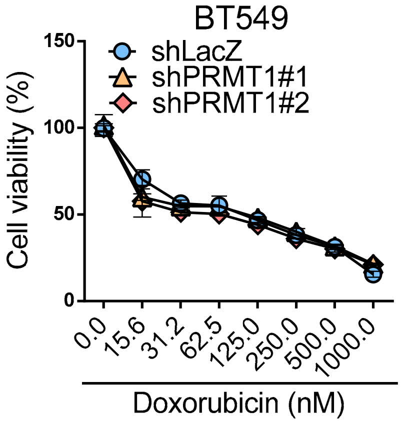

Figure S1. Cell viability of BT549 cells in response to doxorubicin. BT549/shLacZ and shPRMT1 cells were treated with various doses of doxorubicin (0-1000 nM) for 72 hours. Cell viability was measured by 3-(4,5-dimethylthiazol-2-yl)-2,5-diphenyltetrazolium bromide solution (MTT) assay. Data are expressed as a percentage of the control. Values are expressed as the mean ± standard error.
